# Effects of DNA supercoiling on chromatin architecture

**DOI:** 10.1007/s12551-016-0210-1

**Published:** 2016-07-02

**Authors:** Samuel Corless, Nick Gilbert

**Affiliations:** MRC Human Genetics Unit, Institute of Genetics and Molecular Medicine, University of Edinburgh, Crewe Road, Edinburgh, EH42XU UK

**Keywords:** DNA supercoiling, Protein–DNA, Gene regulation, Eukaryotic chromatin, Genome architecture

## Abstract

Disruptions in chromatin structure are necessary for the regulation of eukaryotic genomes, from remodelling of nucleosomes at the base pair level through to large-scale chromatin domains that are hundreds of kilobases in size. RNA polymerase is a powerful motor which, prevented from turning with the tight helical pitch of the DNA, generates over-wound DNA ahead of itself and under-wound DNA behind. Mounting evidence supports a central role for transcription-dependent DNA supercoiling in disrupting chromatin structure at all scales. This supercoiling changes the properties of the DNA helix in a manner that substantially alters the binding specificity of DNA binding proteins and complexes, including nucleosomes, polymerases, topoisomerases and transcription factors. For example, transient over-wound DNA destabilises nucleosome core particles ahead of a transcribing polymerase, whereas under-wound DNA facilitates pre-initiation complex formation, transcription factor binding and nucleosome core particle association behind the transcribing polymerase. Importantly, DNA supercoiling can also dissipate through DNA, even in a chromatinised context, to influence both local elements and large chromatin domains. We propose a model in which changes in unconstrained DNA supercoiling influences higher levels of chromatin organisation through the additive effects of DNA supercoiling on both DNA-protein and DNA-nucleosome interactions. This model links small-scale changes in DNA and chromatin to the higher-order fibre and large-scale chromatin structures, providing a mechanism relating gene regulation to chromatin architecture in vivo.

## Introduction

**Fig. 1:**
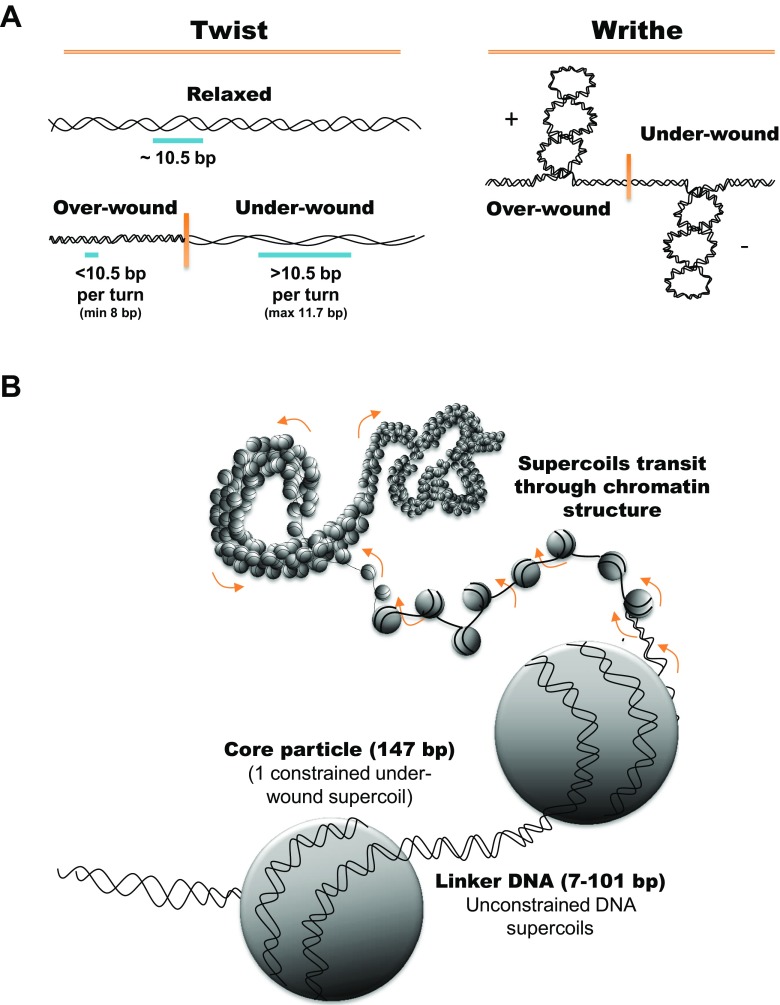
DNA supercoils in chromatin. **a** Twist and writhe in naked DNA. Twist is a change in the number of base pairs per turn of the DNA double helix (*blue bar*). The minimum/maximum (*min*/*max*) values represent the highest level of over-/under-wound DNA twist possible before a forced DNA structural transition (Bryant et al. 2003). Writhe is a structural transition to a coiled helix which has a positive writhe (*+*) for over-wound DNA and a negative writhe (*−*) for under-wound DNA. *Orange bars* represent a barrier to the spread of DNA supercoiling. **b** The basics of chromatin structure. In eukaryotes DNA is bound by nucleosome core particles, interspersed by linker DNA, that form nucleosome arrays. These nucleosome arrays fold into a higher-order fibre and large-scale chromatin structures. DNA supercoiling can transmit through chromatin (*orange arrows*) to influence genome structure and regulation

Supercoiling is a transition from the relaxed state of the DNA double helix to one that is more under- or over-wound (Fig. 1a). In DNA-only systems the presence and influence of supercoiling is discussed using the mathematical concepts of twist and writhe (reviewed in Bates and Maxwell 2005), which are distinct but inter-changeable structural transitions that deform the DNA through changes in the number of base pairs per turn of the helix or through the formation of a coiled helix structure (Fig. 1a). In the context of eukaryotic chromatin, a huge and complex macro-molecular structure of DNA and protein interactions (Fig. 1b), the concepts of twist and writhe as distinct structural entities becomes less clear. In part this is because most of the DNA in eukaryotes is bound to nucleosome core particles, each of which constrain an under-wound DNA supercoil (Fig. 1b). The unconstrained linker DNA has the capacity to form structural transitions (Fig. 1a), but it is relatively short (11–101 bp) (Van Holde 1989) and its capacity to form writhe is uncharacterised (Box 1).

**Fig. 2:**
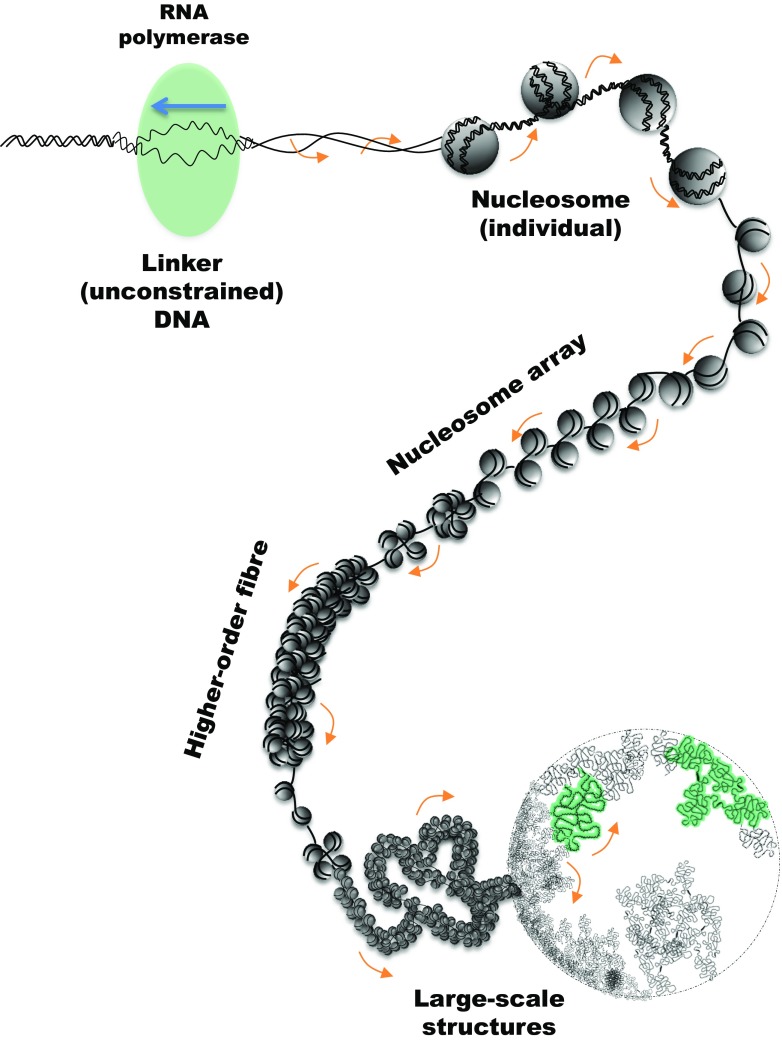
DNA supercoiling influences protein–DNA interactions at different scales of chromatin organisation.* Orange arrows* Dissipating supercoils. Importantly, the limit of supercoil influence is orchestrated by the properties of the higher-order and large-scale chromatin fibres

Nucleosome core particles connected by linker DNA are the fundamental unit of chromatin structure (Fig. 1b). Arrays of core particles form the classical beads-on-a-string-structure (Thoma et al. 1979), which further coils and folds to form higher levels of chromatin organisation (Fig. 2). Each level of chromatin organisation is believed to be an important component of gene regulation—with individual core particles influencing local sequence accessibility, chromatin fibre structure influencing accessibility to a longer region of sequence and large-scale decondensation increasing accessibility over tens to hundreds of kilobases (Bickmore and Van Steensel 2013; Cairns 2009; Gilbert et al. 2004). Mounting evidence supports a role for DNA supercoiling in the structure and regulation of the chromatin fibre, with changes at the nucleosome level being transmitted through the DNA to influence higher levels of organisation.

In eukaryotes most DNA supercoiling is generated by the transcription machinery (Liu and Wang 1987; Ma and Wang 2014), supporting a model where DNA supercoiling at the gene level can influence chromatin organisation immediately around the transcribing polymerase (Sheinin et al. 2013; Teves and Henikoff 2014; Teves et al. 2014), at a distance of several kilobases (Kouzine et al. 2008, 2013a; Naughton et al. 2013a) and over large-scale domains (Naughton et al. 2013a). Furthermore, abortive transcription or the transcription of neighbouring genes generates supercoiling that may prime the chromatin structure of a promoter for subsequent full-length transcription (Meyer and Beslon 2014; Naughton et al. 2013b). In this review we will outline the influence of DNA supercoiling on protein–DNA interactions at different scales to illustrate how changes in supercoiling at the nucleosome level can regulate general principles of chromatin architecture and gene regulation.

## Supercoils influence DNA–protein interactions in linker DNA

Linker DNA forms the smallest unit of influence for unconstrained DNA supercoiling in eukaryotic chromatin (Fig. 2a). It has closest similarity to naked DNA in the chromatin context, but in general it is present only as small stretches of 11–101 bp, which are often contacting linker histones (Van Holde 1989). Longer linker lengths are observed at specific sites when nucleosomes are evicted/moved by other proteins either transiently, in order to allow the binding of other proteins, or more stably through the formation of nucleosome-depleted regions at active promoters and enhancers (Clapier and Cairns 2009; Segal et al. 2006; Struhl and Segal 2013). Importantly, linker DNA can accommodate unconstrained DNA supercoiling which introduces free-energy into the helix with the potential to influence DNA conformation and protein–DNA interactions (Bates and Maxwell 2005). Most of the linker DNA in eukaryotes is torsionally relaxed (Sinden et al. 1980); however small- and large-scale domains of unconstrained DNA supercoiling have been identified in vivo using a psoralen probe of DNA twist (Anders et al. 2014; Bermúdez et al. 2010; Jupe et al. 1993; Kouzine et al. 2013a; Ljungman and Hanawalt 1992, 1995; Matsumoto and Hirose 2004; Naughton et al. 2013a; Teves and Henikoff 2014). An upper estimate of the extent of unconstrained under-wound DNA in chromatin in vivo has been determined to be ~11.29 bp per turn (σ = −0.07) (Box 1). Crucially, this level of supercoiling is more than sufficient to drive DNA to conformations other than the canonical double helix (Irobalieva et al. 2015; Kouzine et al. 2008).

**Fig. 3:**
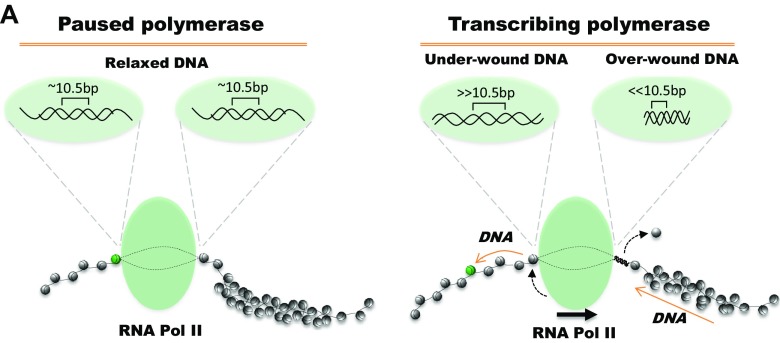
Generating DNA supercoils in chromatin. **a** Transcription by RNA polymerase generates DNA supercoiling by the twin-domain model. In the transition from paused to active transcription the DNA transitions from relaxed (*left panel*) to generating over-wound DNA ahead of the transcription complex (facilitating nucleosome eviction) and under-wound DNA behind the polymerase complex (facilitating nucleosome deposition) (*right panel*)

DNA supercoiling is generated by direct protein–DNA interactions and protein catalytic activity on the DNA double helix (Bates and Maxwell 2005), so whilst not strictly a process that occurs on linker DNA, supercoil generation happens on an unconstrained template in chromatin. In eukaryotes, the most potent generator of DNA supercoils is transcription by RNA polymerase (Fig. 3). The large polymerase complex, greater than 2 MDa (He et al. 2013), has a frictional drag that prevents rotation with the tight helical pitch of the DNA (Liu and Wang 1987; Nelson 1999). The DNA strands are instead twisted by processing polymerase, generating over-wound DNA ahead of the transcription machinery and under-wound DNA behind, known as the twin supercoil domain model (Fig. 2a). Initially a theoretical proposition (Liu and Wang 1987), the validity of this model has now been confirmed in vitro and on chromatinised templates in vivo (Nelson 1999). Similarly, DNA polymerases generate over-wound DNA ahead of the replication fork (Postow et al. 2001) and may generate under-wound DNA on the newly synthesised leading strand (Kurth et al. 2013); however replication has not been demonstrated to form or remodel DNA supercoil distribution in vivo and will not be discussed further in this review. In addition to polymerases, small amounts of DNA supercoiling can be introduced by the association or dissociation of DNA binding proteins that constrain DNA supercoils—for example, nucleosome core particles (Finch et al. 1977; Luger et al. 1997). However, it is generally accepted that the remodelling/removal of core particles is not the major factor regulating unrestrained DNA supercoiling in the linker DNA, as transcription generates 19 under- and 19 over-wound DNA supercoils (one under- and over-wound supercoil every ~10.5 bp) for every under-wound supercoil introduced by the loss of a core particle (~200 bp) (Finch et al. 1977; Liu and Wang 1987), and there is little evidence of large-scale loss of nucleosomes from most actively transcribed regions (Chang et al. 2014; Struhl and Segal 2013). Therefore, in eukaryotes the vast majority of DNA supercoils are believed to be introduced in a transcription-dependent manner.

DNA supercoiling is relieved by the binding and catalytic activity of topoisomerase proteins in unconstrained (linker) DNA (Champoux 2001; Wang 2002). Eukaryotes contain two types of topoisomerase to relieve torsional stress, topoisomerase I which transiently nicks the DNA and relieves supercoils by rotating the nicked strand around the intact strand and topoisomerase II which introduces a double-strand break through which a second intact DNA strand is passed. Topoisomerase I has emerged as the major relaxase for transcription generated DNA supercoiling, enriched at transcriptionally active genes (Baranello et al. 2016; Christensen et al. 2004; Durand-Dubief et al. 2010; Filion et al. 2010; Gilmour et al. 1986), at active under-wound DNA supercoil domains (Naughton et al. 2013a) and particularly in the body of long genes that generate the highest level of DNA supercoiling per full-length transcript (King et al. 2013). In addition, there is evidence to support a role for topoisomerase II at some highly expressed (Kouzine et al. 2013a) or developmentally regulated (Lyu et al. 2006; Sano et al. 2008) genes, although these proteins are most enriched in gene-poor regions (Naughton et al. 2013a; Sano et al. 2008). Steady state DNA supercoiling in unconstrained linker DNA is the result of a dynamic coupling of DNA supercoil introduction/release, and the disruption of either process substantially alters the distribution of unconstrained DNA supercoiling in vivo (Bermúdez et al. 2010; Kouzine et al. 2013a; Matsumoto and Hirose 2004; Naughton et al. 2013a).

**Fig. 4:**
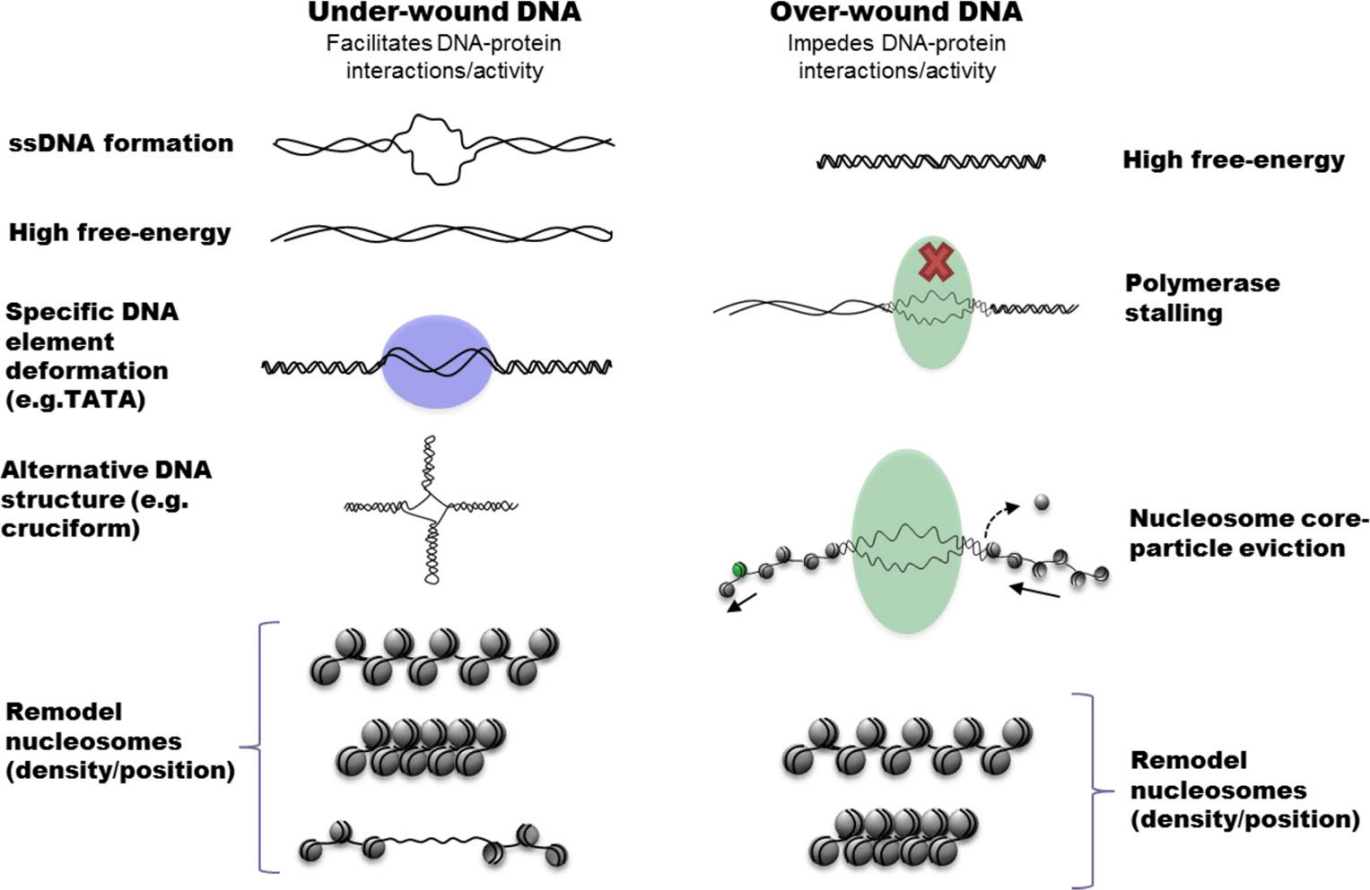
DNA supercoils influence DNA–protein interactions and catalytic activity. An overview of the ways over- and under- wound DNA can influence DNA structures, protein–DNA interactions and the catalytic activity of DNA binding proteins.* ssDNA* Single-strand DNA

The presence of unconstrained supercoiling in the linker DNA introduces free energy into the double helix (Fig. 4) which promotes protein–DNA interactions and protein catalytic activity. Unconstrained DNA supercoils must change the structure of the canonical B-form DNA through a transition in twist, writhe, melted DNA or alternative DNA structures (Figs. 1a, 4). Over-wound DNA generated ahead of polymerases can change the twist of the DNA to give a tighter DNA helix, whereas under-wound DNA changes the twist to give a looser DNA helix (Fig. 1a). Writhe formed on over- or under-wound DNA has a similar structural appearance (Fig. 1a); however the cross-over points are of the opposite handedness so structures will coil differently. Significantly, under-wound DNA free energy can be focussed on sequences with a particular susceptibility to DNA melting, or to the formation of alternative DNA structures, to give targeted structural transitions with regulatory potential (Fig. 4) (Brázda et al. 2011; Kouzine et al. 2008, 2013b; Zhabinskaya and Benham 2011, 2012). The susceptibility of a DNA sequence to melting is determined by the thermodynamic properties of the helix (Zhabinskaya et al. 2015), and promoters and regulatory elements have been identified in vivo that have structures susceptible to DNA supercoiling (Kouzine et al. 2008, 2013a, b; Naughton et al. 2013a; Teves and Henikoff 2014). Recent genome-wide analysis suggests that promoter melting in unconstrained DNA is a general mechanism for the activation of some genes and that this melting occurs preferentially at regions of high susceptibility to DNA supercoiling (Kouzine et al. 2013b). In addition to DNA melting, under-wound DNA can stabilise a transition to alternative DNA structures, including G-quadruplexes, cruciform DNA, triplex DNA, Z-DNA and R-loops. Mapping of alternative DNA structures in vivo has identified an enrichment in important regulatory regions, including promoters, enhancers, replication origins and telomeres, supporting a functional role in chromatinised eukaryotic genomes (Besnard et al. 2012; Biffi et al. 2013; Brázda et al. 2011; Gellibolian et al. 1997; Ginno et al. 2013; Kanoh et al. 2015; Lipps and Rhodes 2009; Moye et al. 2015; Rich and Zhang 2003). Together, these observations indicate that DNA structure is particularly susceptible to under-wound DNA and that changes in the structure of DNA in the linker region could provide an altered high-energy substrate for protein binding (Fig. 4).

### Linker DNA–protein interactions

Understanding how the free energy of DNA supercoiling influences DNA–protein interactions through changes in DNA structure is key to identifying the effects of DNA supercoiling on chromatin architecture. Protein–DNA interactions that require changes to the structure of the DNA double helix, including melting and bending, are generally facilitated by an under-wound DNA structure (Bates and Maxwell 2005). In the context of chromatin structure this includes the nucleosome core particle, the transcription complex, topoisomerase proteins and transcription factors.

#### The nucleosome core particle

The nucleosome core particle has a strong preference for under-wound DNA, with nucleosomes preferentially forming on under-wound DNA plasmids when core histones are incubated in the presence of both over- and under-wound DNA templates (Clark and Felsenfeld 1991). The histone core particle constrains a single under-wound DNA supercoil (Finch et al. 1977; Luger et al. 1997; Richmond and Davey 2003); therefore the binding of a core particle to DNA introduces a compensatory over-wound supercoil into the unconstrained DNA linker. Despite the thermodynamic cost of introducing additional over-wound DNA supercoils, nucleosomes can form on positively supercoiled DNA when it is the only available substrate (Clark and Felsenfeld 1991). However these nucleosomes have a less stable structure, both with respect to histone contacts within the core particle and core particle contacts with the DNA (Clark and Felsenfeld 1991; Gupta et al. 2009; Sheinin et al. 2013; Teves et al. 2014). Surprisingly, mapping nucleosomes on supercoiled and relaxed plasmids in vitro identified no change in nucleosome position, suggesting that supercoiling per se may not strongly influence nucleosome spacing in the fibre (Patterton and Von Holt 1993). Therefore, nucleosome stability but not nucleosome distribution is strongly influenced by the degree of supercoiling on an unconstrained DNA template.

#### The transcription complex

Transcription is strongly influenced by the presence of DNA supercoils in an unconstrained DNA template. Studies in vitro, in prokaryotes and in eukaryotes, have all shown that transcription is more efficient on under-wound DNA templates (Dunaway and Ostrander 1993; Ma et al. 2013; Tabuchi and Hirose 1988; Weintraub et al. 1986). The formation of the pre-initiation complex by the interaction of polymerase complex proteins with promoter DNA is the first step facilitated by the presence of under-wound DNA in vivo, and transcription initiation has been shown to be the key step regulated by DNA supercoiling in the supercoil-sensitive *Bombyx mori* fibroin gene (Tabuchi and Hirose 1988).

In addition to facilitating DNA–protein interactions at the promoter, under-wound DNA can also destabilise double-stranded DNA to promote transcription initiation (Hirose and Suzuki 1988; Kim et al. 2000; Kouzine et al. 2013b). This can be achieved through a local enrichment of unconstrained under-wound DNA, as observed at most eukaryotic gene promoters (Kouzine et al. 2013a; Naughton et al. 2013a; Teves and Henikoff 2014), or by the general transcription factor TFIIH which uses a translocase mechanism to generate under-wound DNA and “open” the promoter (Kim et al. 2000).

Once the transcription complex is bound and initiated, DNA supercoiling also influences the translocation of polymerase between the strands of the DNA double helix. Under-wound DNA is more efficiently transcribed, with in vitro studies demonstrating an increased transcription rate and a decreased pause frequency and duration (Ma et al. 2013). Conversely, over-wound DNA has a lower transcription rate and, at high levels, prevents the processivity of the polymerase complex because the tight DNA helix prevents DNA strand separation. In prokaryotes it has been demonstrated that over-wound DNA ahead of the polymerase complex leads to polymerase pausing and that release of this superhelical tension by topoisomerase regulates transcriptional bursting (Chong et al. 2014). More recently, Baranello et al. (2016) proposed a similar mechanism for transcription pausing in human cells.

Key steps of transcription are regulated by DNA supercoiling; this is of particular importance as transcription generates the under-wound promoter environment which facilitates the binding of subsequent transcription complexes, the opening of the promoter region and polymerase catalytic activity. The observation that human promoters generate high levels of abortive divergent transcription prior to transcribing a full-length coding transcript (Core et al. 2008; He et al. 2008; Preker et al. 2008; Seila et al. 2009) led us to propose that the function of abortive transcription at the promoter is to generate an under-wound DNA environment to facilitate efficient transcription (Naughton et al. 2013b). We suggest that the generation of DNA supercoiling is targeted to promoter regions to facilitate polymerase complex protein–DNA interaction and catalytic activity.

#### Topoisomerases

Topoisomerase activity is also influenced by DNA supercoiling—in particular the transcription associated topoisomerase I. Topoisomerase I preferentially binds supercoiled DNA (Madden et al. 1995; Muller 1985) and is highly enriched at transcriptionally active regions of the genome (Baranello et al. 2016; Durand-Dubief et al. 2010; Filion et al. 2010; Gilmour et al. 1986; King et al. 2013; Naughton et al. 2013a). Furthermore, the catalytic activity of topoisomerase I is critically regulated by the presence of unconstrained DNA supercoils (Koster et al. 2005), as well as by RNA polymerase modifications (Baranello et al. 2016). Topoisomerase I clamps tightly around the DNA, cuts one strand of the DNA double helix and rotates it around the intact strand using the free energy of unconstrained DNA supercoiling (Champoux 2001; Koster et al. 2005; Leppard and Champoux 2005). This mechanism releases a single supercoil per revolution of the helix, with multiple revolutions occurring between strand break and religation in a torsion-force dependent manner (Koster et al. 2005). This controlled-rotation mechanism relaxes over- and under-wound DNA supercoils, although there is some evidence that the relaxation of over-wound DNA supercoils is more efficient in vitro (Frøhlich et al. 2007), possibly accounting for the observed enrichment of under-wound DNA at transcriptionally active regions in vivo, although this aspect requires further investigation.

The relationship between eukaryotic topoisomerase II and DNA supercoiling is less well understood. In terms of the relaxation of transcription-derived supercoils, only topoisomerase IIβ is expressed throughout the cell cycle (Woessner et al. 1991). However, neither the protein binding or catalytic activities of topoisomerase IIβ seem to be directly influenced by DNA supercoiling (McClendon et al. 2005). Instead the relationship between topoisomerase II, DNA supercoiling and catalytic activity may be more indirect, promoting the untangling (rather than further entanglement) of DNA during decatenation, the removal of DNA writhe or the regulation of DNA supercoils from the base of chromatin loops (Nitiss 2009).

#### Transcription factors

The most interesting—but least studied—potential for DNA supercoiling in unconstrained DNA is to promote the binding of transcription factors through changes in DNA structure that alter protein binding at specific sites. The clearest in vivo example occurs at the far upstream element (FUSE) of the human *c-myc* gene, a sequence located 1.5 kb upstream of the promoter that melts to single-stranded DNA in a transcription-generated supercoil-dependent manner (Kouzine et al. 2008). Following the supercoil-dependent change in DNA structure, the FUSE binding proteins (FBP) and FUSE interacting repressor (FIR) bind to the FUSE element and regulate subsequent gene activation. Transcription inhibition or release of DNA supercoils by nicking the DNA return the DNA structure to the double-stranded form, and it no longer binds FBP or FIR proteins. A similar mechanism has been proposed at the* USP29* gene (Liu et al. 2011), but this locus requires further functional characterisation.

At promoter regions an under-wound DNA structure may facilitate the formation of a disrupted, alternative or melted DNA structure that influences DNA protein binding. The TATA-box DNA sequence element is predicted to be sensitive to under-wound DNA supercoiling, and the structure of TATA-box binding protein bound to DNA identifies that the DNA has an under-wound structure (Kim et al. 1993; Tabuchi et al. 1993). It has therefore been proposed that TATA-elements are DNA supercoil sensitive. Furthermore, alternative DNA conformations stabilised by under-wound DNA, including G-quadruplexes and Z-DNA, have been demonstrated to form in vivo, and each have specific binding proteins (Kanoh et al. 2015; Rich and Zhang 2003). Finally, the results of recent work aimed at mapping single-stranded DNA in vivo provide a rich resource for identifying elements that melt in a supercoil-dependent manner and suggest that human and mouse gene promoters are particularly susceptible to DNA melting (Kouzine et al. 2013b). Together, these results show a correlation between the presence of DNA structures sensitive to DNA supercoiling, regions shown to have an under-wound DNA structure and proteins that bind these DNA structures. However, direct experimental evidence linking these three properties is limited to the FUSE element, and further experimentation is necessary to determine general principles linking these factors.

## Supercoils influence DNA–protein interactions in a nucleosome array

We have so far considered the role of DNA supercoiling in the context of unconstrained DNA, which for the purposes of discussion was considered to behave as naked DNA; however in reality ~80 % of the DNA in eukaryotic genomes is bound to nucleosome core particles (Figs. 1b, 2) (Wolffe 1998; Zlatanova et al. 2009). Nucleosomes are formed from a H3–H4 tetramer and two dimers of H2A–H2B proteins (the core particle) which wrap 147 bp of DNA ~1.8 times in a left-handed coil around their outer surface. DNA wrapping around the core particle causes the DNA to adopt a writhed structure, and each nucleosome in the genome constrains a single under-wound supercoil. The association of nucleosomes with DNA produces a significant barrier to transcription in vitro, and the first nucleosome downstream of the initiation site generally acts as a barrier to polymerase progression, leading to pausing in vivo (Kulaeva et al. 2013). In addition, the association of core particles with DNA occludes transcription factor binding sites and prevents the formation of melted DNA and alternative DNA structures at regions that interact with core histones (Segal et al. 2006; Struhl and Segal 2013). Occluded sequences can only be exposed for protein–DNA interaction by nucleosome remodelling or eviction, moving the sequence from a ‘covered’ position to an accessible position in the linker DNA. Importantly, nucleosome eviction would also introduce additional under-wound DNA supercoils into the unconstrained DNA which may further facilitate protein binding to the uncovered sequence. Therefore, the nucleosome core particle can regulate the distribution of unconstrained DNA supercoiling on the underlying sequence in order to promote/inhibit other protein–DNA interactions.

### The nucleosome array

Nucleosomes core particles are bound every ~200 bp in eukaryotic genomes, constraining a large amount of under-wound DNA, in addition to unconstrained under-wound DNA in the linker region (Fig. 1b). DNA supercoils impact the stability, distribution and density of core particles and thereby can alter the structure of the nucleosome array (Fig. 2). Although in vitro work by Patterton and Von Holt (1993) showed that DNA supercoiling per se does not alter the position of nucleosomes on a DNA sequence, the sharp transition from one superhelical state to another seems to have a profound influence on the distribution and stability of nucleosome core particles in a nucleosome array (Petesch and Lis 2008; Teves and Henikoff 2014). As discussed previously, the first nucleosome encountered by a transcribing polymerase acts as a barrier and leads to pausing. However, once this first nucleosome is passed, the polymerase proceeds at a rate comparable to that of naked DNA with limited pausing at subsequent core particles (Darzacq et al. 2007; Kwak et al. 2013). One compelling hypothesis is that the free energy of over-wound DNA supercoils ahead of the transcribing polymerase destabilises nucleosome interactions (Clark and Felsenfeld 1991; Sheinin et al. 2013; Teves and Henikoff 2014), providing an optimum nucleosome-free DNA substrate for transcription. As nucleosome core particles constrain under-wound DNA, over-wound DNA may destabilise protein–protein interactions in the core particle and protein–DNA interactions in the nucleosome (Sheinin et al. 2013). The dissociation of the first nucleosome may be hindered by the low level of over-wound DNA generated by a relatively short transcript, but once this barrier is overcome the nucleosomes are disrupted ahead of the advancing polymerase at a faster rate. In the case of highly transcribed genes, core particles are completely dissociated ahead of the transcribing polymerase (Kulaeva et al. 2013; Studitsky et al. 1994), revealing a region of unconstrained (linker) DNA template for transcription. At lower transcription rates, short lengths of DNA transiently dissociate from the core particle ahead of the polymerase and re-associate with the core particle behind the polymerase, allowing the same nucleosome to be deposited behind the transcription machinery (Chang et al. 2014; Kulaeva et al. 2013; Studitsky et al. 1994). In both cases the over-wound DNA ahead of the transcription machinery can destabilise core particle interactions and the under-wound DNA behind the transcription machinery can promote the reformation of the nucleosome array (Clark and Felsenfeld 1991). In this way the influence of supercoiling on protein–DNA interactions, which we discussed previously in the context of unconstrained linker DNA, can also influence the first level of chromatin fibre organisation.

In addition to remodelling nucleosome array structures through the body of a gene, DNA supercoiling is proposed to have a role in remodelling chromatin structure at gene promoters (Naughton et al. 2013b). Gene promoters are generally under-wound in eukaryotes (Kouzine et al. 2013a; Naughton et al. 2013a; Teves and Henikoff 2014) and, in the case of humans, many promoters are divergently transcribed so that each transcript generates under-wound DNA that is focused onto the promoter region (Core et al. 2008). Furthermore, recent advances in RNA sequencing technology have shown that most transcripts are aborted after ~50 bp and that most transcription which occurs at promoters is non-protein coding and often rapidly degraded (Core et al. 2008; Kwak et al. 2013; Scruggs et al. 2015). We have previously proposed that divergent transcription sets up a chromatin environment that facilitates full-length gene expression through transcription factor binding, pre-initiation complex formation and transcription initiation (Naughton et al. 2013b). Early studies also proposed a role for DNA supercoils in the formation of nucleosome-depleted regions found at the promoters of active genes (Villeponteau and Martinson 1987; Villeponteau et al. 1984; Weintraub 1983). Recently this hypothesis has been re-visited by Scruggs et al. (2015) who identify a relationship between divergent transcription and nuclease hypersensitivity and suggest a role for DNA supercoiling in remodelling nucleosomes to expose gene promoters in the unconstrained linker DNA.

Nucleosome remodelling through DNA supercoiling is also a specific mechanism employed by some classes of chromatin remodelling enzymes (Hauk and Berger 2016). Remodellers containing the SNF2p-related ATPase domain have been shown to generate long (hundreds of base pairs) under-wound DNA loops in vitro (Havas et al. 2000; Lia et al. 2006). This mechanism slides a nucleosome along the DNA and produces an unconstrained under-wound template that may promote protein binding specifically in the loop generated by the chromatin remodelling enzyme. The formation of this small-scale domain of unconstrained under-wound DNA and the reversal back to a non-remodelled chromatin fibre both occur in an ATP-dependent manner (Havas et al. 2000), and we speculate that co-factors may specifically recruit this class of remodellers to prime regulatory sequence for supercoil-specific DNA–protein interactions. Other chromatin remodelling complexes, and SNF2 complexes in vivo, have not been fully characterised, but it has been proposed that DNA supercoiling-based remodelling may form a general mechanism for the re-organisation of nucleosome arrays (Lia et al. 2006).

Perhaps the most surprising property of DNA supercoiling in a nucleosome array is that the free energy of supercoils transmits freely through the unconstrained DNA, without being significantly blocked by the presence of nucleosome core particles. This has been demonstrated most convincingly using in vitro single molecule experiments with magnetic and optical tweezers (Lavelle et al. 2010). Using these approaches nucleosome arrays have been shown to reversibly accommodate high levels of under- and over-wound DNA supercoils, with the authors of one study proposing that a role for chromatin is to act as a ‘topological buffer’ (Bancaud et al. 2006). These results suggest that the dissipation of DNA supercoils is not hindered by wrapping DNA around core particles in a nucleosome array; instead they support in vivo observations that DNA supercoiling can transmit through the chromatin over several kilobases and, in combination, over large-scale DNA supercoil domains (Kouzine et al. 2008; Naughton et al. 2013a). In addition, nicking the DNA with bleomycin every few kilobases is sufficient to relax DNA supercoiling in human cells (Naughton et al. 2013a), further supporting that supercoils can transmit freely through the nucleosome array.

In summary, DNA supercoiling transmits through nucleosome arrays and influences core particle stability, position and density in a transcription- (or chromatin remodelling protein) dependent manner. This influences the accessibility of linker DNA, which provides a reservoir of unconstrained DNA supercoils, and the capacity of supercoiling to promote/inhibit DNA–protein interactions.

## Supercoils influence high levels of chromatin organisation

### Higher-order chromatin fibre

The next level of chromatin organisation above the nucleosome array is the higher-order fibre (Fig. 2), which is proposed to regulate the accessibility of linker DNA through changes in the regularity/disruption of the fibre structure. The structure of the higher-order fibre is controversial (Fussner et al. 2011; Maeshima et al. 2010; Staynov 2008), although in vitro observations by electron microscopy and crystallography indicate that the predominant folding is a 30-nm fibre arranged in a solenoid or zig-zag helical structure (Kruithof et al. 2009; Schalch et al. 2005; Thoma et al. 1979; Wolffe 1998). Beyond the 30-nm fibre higher-order structures are even less well defined, with additional folding and coiling predicted to form ~100-nm chromonema fibres and 200- to 300-nm fibres contributing to large-scale chromatin structures (Bak et al. 1977; Belmont and Bruce 1994; Sedat and Manuelidis 1978; Taniguchi and Takayama 1986).

The higher-order chromatin fibre is influenced by the underlying distribution of nucleosomes on the nucleosome array and by other DNA–protein interactions. A fibre containing regular repeats of the Widom-601 nucleosome positioning sequence has a uniform structure which has been determined by X ray crystallography (Schalch et al. 2005). However, chromatin fibres in vivo are believed to be much more heterogeneous in terms of linker length, stability and the position of nucleosomes. This is particularly true in gene dense/transcriptionally active regions which have high levels of chromatin disruptions caused by transcription, nucleosome remodelling, nucleosome depletion and DNA-binding proteins. DNA supercoiling influences all of these processes, and we propose that transcription-generated supercoils have considerable influence on the structure of the higher-order chromatin fibre, with an under-wound fibre being more disrupted and with more accessible linker DNA.

Previously we showed that disrupted higher-order fibre structures correlate better with gene density than with gene expression in human chromatin (Gilbert et al. 2004). Our proposal that higher-order fibre structure is influenced by transcription-generated DNA supercoiling is in agreement with this finding, as lower expression of many neighbouring genes could have an additive effect on supercoiling and fibre structure, whereas high levels of transcription from a single gene in a gene-poor region may dissipate and dilute the effect of DNA supercoiling. Under these conditions it would be expected that fibre structure would be most affected by the cumulative DNA supercoiling of gene-dense regions. Disruption of the higher-order fibre, caused by changes in the underlying nucleosome array, demonstrate that DNA–protein interactions can have indirect effects on chromatin structure with the potential to regulate accessibility within the chromatin fibre. We propose that changes in nucleosome position/turnover and other protein–DNA interactions, in a DNA supercoil-dependent manner, alter the structure of the higher-order fibre. Through this mechanism active regions of the genome are maintained with an accessible chromatin structure, which further facilitates the association of proteins (supercoil dependent or not) with the unconstrained linker DNA.

### Large-scale chromatin structures

Above the level of the higher-order fibre, chromatin is organised into large-scale domains which partition the genome into structural and regulatory units (Benyajati and Worcel 1976; Dixon et al. 2012; Lupiáñez et al. 2015; Naughton et al. 2013a). Topologically isolated domains of DNA supercoiling were first identified by determining the number of nicks required to fully relax a deproteinised *Drosophila* genome (Benyajati and Worcel 1976). Similar loops were identified by electron microscopy, and the identification of topoisomerase II and condensin at the base of these loops indicated an important role for DNA supercoiling (Earnshaw and Heck 1985; Hirano and Mitchison 1994; Paulson and Laemmli 1977). More recently, our laboratory developed a molecular approach to map DNA supercoil domains in vivo, using a psoralen-based molecular probe of DNA twist, and the results of this mapping study led us to a similar conclusion—that the genome is organised into ~130-kb domains of unconstrained DNA supercoiling (Naughton et al. 2013a). We observe some similarity with the boundaries of larger topological associated domains (TADs) (~900 kb), another large-scale chromatin structure determined by 3C-based proximity ligation methods (Dixon et al. 2012), and suggest that TADs are further organised into smaller supercoiling domains that reflect the local transcriptional environment.

When we measure large-scale DNA supercoil domains we are actually measuring the level of unconstrained DNA supercoiling in the linker DNA of nucleosome arrays, within a higher-order chromatin fibre (Fig. 2). Nicking the DNA every few kilobases is sufficient to release detectable DNA supercoils, and the resulting inhibition of transcription or topoisomerases remodels the distribution of DNA supercoiling over large-scale domains (Naughton et al. 2013a). Together, these results demonstrate that DNA supercoil domains are modifiable and formed by the balanced introduction and relaxation of unconstrained supercoiled DNA.

The large-scale influence of DNA supercoiling on chromatin structure and genome regulation can also be observed cytologically, through changes in the compaction of large-scale chromatin structures. Fluorescence in situ hybridisation studies on human tissue culture cells revealed that under-wound DNA supercoil domains are cytologically decompacted compared to gene-poor over-wound domains and that this decompaction is lost in the presence of transcription inhibitor or DNA-nicking reagents (Naughton et al. 2013a). The results reported by Matsumoto and Hirose (2004) provide further support for the large-scale influence of DNA supercoiling on chromatin structure and gene expression. These authors observed ~150 domains of under-wound DNA in* Drosophila* polytene chromosomes; these correspond to nascent RNA transcription and are lost following nicking with bleomycin or transcription inhibition. When polytene chromosomes are subjected to heat shock, they display a massive chromatin decompaction of the region containing the heat shock protein 70 (HSP70) gene. Measuring under-wound DNA with a biotin psoralen probe and visualising by immunofluorescence with streptavidin-green fluorescent protein, these same authors demonstrated that DNA becomes under-wound prior to expression of the HSP70 gene. This result indicates that transcription-generated DNA supercoiling primes large-scale chromatin domains prior to productive gene expression and together with other results supports a role for unconstrained DNA supercoiling in the structure of large-scale chromatin domains in vivo.

Large-scale DNA supercoil structures are demarcated by unidentified ‘topological isolating factors’. There are prime candidates for this role, including CTCF (CCCTC-binding factor) and condensin (Hirano 2016; Phillips and Corces 2009), but further investigation is required to determine the role of these proteins (if any). Furthermore, it is possible that the boundaries of supercoil domains are not determined by topological insulators, but are instead a reflection of fewer genes contributing to a cumulative enrichment of DNA supercoiling. In this case, supercoils dissipate into non-transcribed chromatin, and the influence of supercoiling on nucleosome arrays and higher-order structure diminishes. Importantly, the boundaries of DNA supercoil domains, whether determined by topological insulators or supercoil diffusion, affect the extent of influence of within-domain DNA supercoils on higher-order fibre structure, nucleosome array structure, linker DNA structure and protein–DNA interactions.

## Perspective

**Fig. 5:**
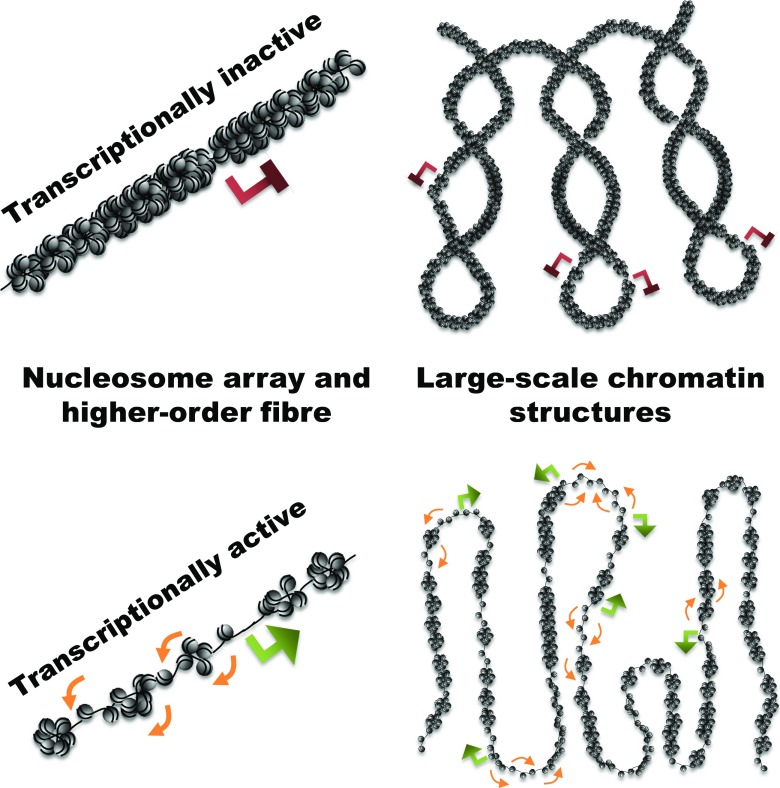
Transcription-generated DNA supercoils influence nucleosome array, higher-order fibre and large-scale chromatin organisation. Transcriptionally inactive chromatin has a compacted fibre structure and cytologically compact large-scale architecture. In contrast, transcriptionally active regions have an under-wound DNA structure that forms a decompacted/disrupted higher-order chromatin fibre and cytologically decompact large-scale chromatin structures. *Green arrows* actively transcribed genes, *red arrows* inactive genes. *Orange arrows* Under-wound DNA supercoils generated upstream of a transcribing polymerase, which are preferentially maintained at promoters and at transcriptionally active large-scale DNA supercoil domains (e.g. Naughton et al. 2013a)

Understanding the influence of DNA supercoiling on chromatin structure and gene regulation is in its infancy, despite almost 30 years of research following the publication of the twin-supercoil domain model (Liu and Wang 1987). It has been demonstrated that DNA supercoiling alters the structure of unconstrained linker DNA (Kouzine et al. 2008; Naughton et al. 2013a), the distribution of core particles in the nucleosome array (Petesch and Lis 2008; Teves and Henikoff 2014) and the decompaction of large-scale chromatin domains (Matsumoto and Hirose 2004; Naughton et al. 2013a). We propose a unified model linking DNA supercoil changes and protein–DNA interactions at the small scale, generated by transcription (and to a lesser extent chromatin remodelling), with changes in higher-order and large-scale chromatin structure (Fig. 5). These changes are orchestrated through DNA supercoil-dependent differences in DNA structure which influence nucleosome position and stability. This altered nucleosome array changes the properties of the higher-order chromatin fibre so that it is more/less disrupted or has an altered helical structure. Changes in the chromatin fibre then influence higher levels of chromatin organisation, which manifest as changes in large-scale chromatin structure.

In this model there is the potential for local amplification of unrestrained DNA supercoiling and gene expression in regions containing multiple active genes (Fig. 5). At the small scale, bidirectional transcription is a common feature of human gene promoters, and we have previously proposed that these abortive non-coding transcripts are generating under-wound DNA to prime local chromatin structure for subsequent full-length transcription (Naughton et al. 2013b). However, the influence of DNA supercoiling on the expression of neighbouring genes has also been inferred at the kilobase scale by linking co-transcriptional regulation to gene orientation (Meyer and Beslon 2014). In this latter study divergent promoters show mutual elevation of expression, as these promoters drive under-wound DNA into their neighbour, whereas convergent promoters show mutual repression which may be due to the presence of over-wound DNA. At the large scale, the observation of ‘transcription ripples’, in which intense transcription of rapidly activated genes subsequently activates nearby genes (Ebisuya et al. 2008), could potentially be explained by DNA supercoiling. In this work the authors show that transcription influences neighbouring genes within ~100 kb domains, promoting gene expression independent of gene orientation. This is highly reminiscent of the DNA supercoil domains we identified in vivo (Naughton et al. 2013a) and we hypothesise that a link between DNA supercoiling and the ‘transcription ripple’ effect will be identified. Together, these observations support a link between gene regulation and proposed/known properties of DNA supercoiling in chromatin. Crucially, future experiments must determine the mechanisms linking DNA supercoiling to gene expression and chromatin structure.

To further understand how DNA supercoiling influences protein–DNA interaction, chromatin structure and gene regulation it is necessary to determine the properties of promoters and regulatory elements that are sensitive to DNA supercoiling. The FUSE element of c-myc has been the classic example of a supercoil sensitive protein-binding sequence element, and the TATA-box sequence has been implicated as supercoil sensitive at promoters, but it is unclear how prevalent supercoil-dependent regulation is in eukaryotic genomes. A recently described single-stranded DNA sequencing technique for mapping melted DNA structure in human cells provides a starting point for understanding the prevalence of supercoil-sensitive sites in the genome (Kouzine et al. 2013b). In addition, generating improved datasets of DNA supercoil distribution using psoralen (Bermúdez et al. 2010; Kouzine et al. 2013a; Naughton et al. 2013a; Teves and Henikoff 2014) will allow a detailed investigation of the relationship between supercoiling, sequence and in vivo melting properties to determine the mechanisms linking supercoiling and gene expression.

Above the scale of individual genes, the importance of DNA supercoiling on chromatin structure in vivo has so far been determined by the addition of transcription inhibitors, topoisomerase inhibitors or DNA-nicking agents or through heat shock in *Drosophila* polytene chromosomes (Kouzine et al. 2013a; Matsumoto and Hirose 2004; Naughton et al. 2013a). In each case it is difficult to separate the influence of DNA supercoiling from the influence of transcription; the latter introduces the majority of DNA supercoiling in eukaryotes but also has important influences on chromatin structure separate from its supercoiling activity. To separate transcription from supercoiling activity it will be important to develop methods which specifically target supercoil-modifying enzymes to particular regions of the genome. For example, the bacterial enzyme DNA gyrase introduces under-wound DNA in a transcription-independent manner (Champoux 2001) and could be tethered to a TAL (transcription activator-like) effector protein (Bogdanove and Voytas 2011) to specifically generate under-wound DNA in a locus of choice. This would allow the determination of the influence of under-wound DNA on chromatin structure and gene expression in a targeted manner in vivo. Topoisomerases with different activities could be used similarly to identify the influence of supercoil relaxation on chromatin structure and gene expression. Finally, these topological modifiers could be targeted to candidate supercoil-sensitive promoters to tease apart the precise mechanisms of supercoil regulation at candidate genes.

Taking these approaches to understand how transcription and DNA sequence function together, through DNA supercoiling, to facilitate protein binding and chromatin structure will offer fresh insight into the role of DNA structure in the chromatin fibre. The model we propose identifies DNA supercoiling as a key factor regulating general principles of chromatin architecture, in addition to individual protein–DNA interactions, by transmitting information about gene regulation from the site of transcription through the chromatin fibre and over large-scale domains.

**Table:** 

Box 1
Unconstrained supercoils in DNA have the capacity to induce changes in twist and/or writhe (Fig. 1a), which are transitions from a relaxed double helix (~10.5 bp/turn, no writhe) to one that stores free energy as a change in the number of turns of the helix per nucleotide (twist) (under-wound <10.5 bp/turn, over-wound >10.5 bp/turn) or in the formation of a coiled-helix or superhelix (writhe). These transitions in DNA structure may influence DNA-binding proteins, but the relative importance of these structural changes is uncharacterised in chromatin. To infer the importance of twist/writhe it is important to establish the supercoiling density (σ) that is expected to occur within chromatin and to establish the biophysical limitations of DNA at this supercoil state. Supercoil density (σ) is determined by calculating the change in the number of times one strand of DNA crosses the other between a relaxed and supercoiled state (reviewed in Bates and Maxwell (2005)). An upper estimate of unconstrained under-wound DNA in human chromatin was determined to be the equivalent of 11.3 bp/turn (σ = −0.07) in a writhe-free system (Kouzine et al. 2008). Using a Cre recombinase system the authors’ excised DNA minicircles containing a footprint of in vivo DNA supercoiling from a region between inducible, highly expressed and divergent promoters. At supercoiling densities slightly below this level, Boles et al. (1990) determined by electron microscopy that the contribution of twist and writhe in naked DNA has a ratio of 1:2. The maximum levels of twist that DNA can withstand before forcing a structural transition was determined by Bryant et al. (2003) using a force-measuring optical trap under conditions that preclude the formation of writhe. In this system the DNA can withstand a remarkable amount of twist, up to 11.7 bp/turn (σ = −0.1) for under-wound DNA and 8.0 bp/turn (σ = 0.32) for over-wound DNA. Therefore, DNA can accept significant twist and writhe, the balance of which is determined by the level of tension in the system. A further complicating factor influencing the distribution of twist and writhe in the unconstrained DNA of chromatin could result from the relatively short length of linker DNA (7–101 bp) and the formation of higher-order chromatin fibres with interactions between proteins in adjacent regions of the fibre (Van Holde 1989; Wolffe 1998). Therefore, we can infer that the distribution of these properties probably falls somewhere between naked DNA in solution (1:2 twist:writhe) and naked DNA under tension (1:0 twist:writhe), with the true distribution influenced by supercoil density and local properties of the chromatin fibre.
